# Green biosynthesis of titanium dioxide nanoparticles incorporated gellan gum hydrogel for biomedical application as wound dressing

**DOI:** 10.3389/fchem.2025.1560213

**Published:** 2025-04-17

**Authors:** Yongtao Su, Xianwei Zhu, Guangqi Xu, Zhongzheng Guan, Wei Jiao, Zhixin Zhang, Yifei Sun, Chunlei Wang, Rong Zhang, Qianqian Luo, Ying Sui, Mahani Yusoff, Mohd Hasmizam Razali

**Affiliations:** ^1^ College of Pharmacy, Institute of Tissue Regeneration and Wound Repair, Shandong Traditional Chinese Medicine University, Jinan, China; ^2^ Department of Wound Repair and Plastic Surgery, Affiliated Hospital of Shandong University of Traditional Chinese Medicine, Jinan, China; ^3^ Department of Burn skin surgery, PKU Care Luzhong Hospital, Zibo, China; ^4^ Department of Rehabilitation, Shandong First Medical University Affiliated Occupational Disease Hospital (Shandong Provincial Occupational Disease Hospital), Jinan, China; ^5^ Morphology Laboratory, Binzhou Medical University, Yantai, China; ^6^ Faculty of Bioengineering and Technology, Universiti Malaysia Kelantan, Kota Bharu, Kelantan, Malaysia; ^7^ Faculty of Science and Marine Environment, Universiti Malaysia Terengganu, Kuala Terengganu, Terengganu, Malaysia

**Keywords:** biosynthesis, nanomaterial, hydrogel, biomaterial, tissue engineering

## Abstract

Titanium dioxide nanoparticles (TiO_2_NPs) are widely synthesized chemically for industrial applications. However, these methods often have negative environmental impacts, rendering them unsuitable for biomedical applications. Green synthesis approaches offer a promising alternative due to their simplicity, environmental friendliness, and cost-effectiveness. In this study, we report the biosynthesis of TiO_2_NPs using Morus alba leaf extract and their subsequent incorporation into a gellan gum (GG) biopolymer to create a hydrogel. The physicochemical properties of the biosynthesized TiO_2_NPs and the TiO_2_NP@GG hydrogel were characterized using Fourier-transform infrared spectroscopy (FTIR), X-ray diffraction (XRD), scanning electron microscopy (SEM), energy-dispersive X-ray spectroscopy (EDS), and X-ray photoelectron spectroscopy (XPS). Furthermore, the bioactivity of the materials was investigated through antibacterial assays against *Staphylococcus aureus* and *Escherichia coli*, as well as *in vitro* wound healing studies using a 3T3 fibroblast scratch assay. XRD analysis confirmed the successful formation of anatase phase TiO_2_. SEM images revealed the presence of irregular and rod-shaped TiO_2_ nanoparticles, with EDS analysis confirming their composition of oxygen and titanium. The particle size was determined to be 80–90 nm, and the nanoparticles exhibited homogeneous distribution throughout the gellan gum biopolymer network. The TiO_2_NP@GG hydrogel displayed significant antibacterial activity against both *S. aureus* and *E. coli*. *In vitro* wound healing studies using a scratch assay on 3T3 fibroblast cells seeded onto the hydrogel demonstrated a high cell survival rate and enhanced cell migration, suggesting potential for biomedical applications as a wound dressing material.

## Introduction

Nanomaterials have become a central focus within the swiftly expanding realm of nanobiotechnology, a discipline that combined material science, chemistry, physics, biotechnology, and nanotechnology at a nanoscale level (Chaudhary et al., 2024; Abid et al., 2024; Mittal et al., 2024). An essential aspect of this domain is the endeavor towards creating secure, environmentally sustainable, and sanitary approaches for producing nanoparticles (Irede et al., 2024). Green synthesis approaches are gaining significant traction due to their potential applications in generating environmentally friendly materials for diverse purposes, including antiviral and antibacterial agents, diagnostics, renewable materials, targeted drug delivery systems, environmentally responsive solvents, and anticancer agents (Mohammed et al., 2024; Abuzeid et al., 2023; Bhatt and Saraswat, 2024; Nag et al., 2024). Conventional chemical synthesis methods often produce larger particles and may involve hazardous processes, leading to side effects like increased agglomeration and reduced nanoparticle stability (Saxena et al., 2012; Taleb et al., 1997; Mukherjee et al., 2001). Consequently, developing nature-friendly synthesis techniques capable of producing well-dispersed, stable nanoparticles with controllable sizes and minimal energy consumption is crucial.

Titanium dioxide nanoparticles (TiO_2_NP) is a versatile semiconducting transition metal oxide material. Its ease of control, non-toxicity, cost-effectiveness, and resistance to chemical corrosion make it suitable for various applications such as chemical sensors, solar cells, and environmental purification processes (Khan et al., 2002). TiO_2_NP possesses diverse and unique optical, magnetic, and electrical properties compared to other materials (Xu et al., 2008). It exists in both crystalline and amorphous forms, with three main polymorphous crystalline structures: brookite, rutile, and anatase (Mahshid et al., 2007). Conventional synthesis methods for TiO_2_NP pose significant environmental challenges that extend beyond immediate hazards. These methods, which often involve high temperatures, strong acids or bases, and organic solvents, contribute to considerable energy consumption and a large carbon footprint, exacerbating climate change. They also generate toxic byproducts and wastewater that can lead to water pollution and long-term ecological damage if not properly managed (Priya et al., 2024). The reliance on non-renewable resources in these methods exacerbates resource depletion, while the potential release of nanoparticles into the environment raises concerns about bioaccumulation and its impact on ecosystems and human health. Additionally, the occupational hazards associated with handling toxic chemicals in conventional synthesis processes pose risks to workers (Schulte et al., 2013). The shift to green synthesis methods, which utilize renewable resources, operate under milder conditions, and produce minimal hazardous waste, offers a more sustainable alternative (Soltys et al., 2021). These methods mitigate environmental and health impacts by reducing energy use, minimizing waste, and employing non-toxic reagents, aligning with stricter regulatory guidelines and promoting sustainable practices (Larrañaga-Tapia et al., 2024). Thus, the urgency to adopt green alternatives is driven by the need to address the comprehensive environmental and health impacts associated with conventional TiO_2_NP synthesis methods. Ultimately, the move toward green synthesis of TiO_2_NP is not just a matter of compliance or reducing hazards; it is a critical step in reducing the overall environmental impact of nanotechnology, preserving natural resources, and protecting both human health and ecological systems from the potentially harmful effects of conventional manufacturing processes. Table 1 presents the existing methods to synthesis TiO_2_NP, their advantages, disadvantages, polymorph of synthesized TiO_2_NP and its application.

**TABLE 1:** Summary of existing methods for synthesizing TiO_2_NP including their advantages, limitations, polymorphs, and applications.

TiO2NP polymorph synthesized	Synthesis method	Advantages and disadvantages	Application	Ref.
Anatase, Rutile	Sol-gel	Pros High purity, control over particle size and morphology Cons Requires high-temperature calcination, long processing time	Photocatalysis	Khan (2025)
Anatase, Rutile	Hydrothermal	Pro High crystallinity, uniform particle size, environmentally friendly Cons Requires specialized equipment, high energy consumption	Energy storage	Si et al. (2023)
Anatase, Rutile	Solvothermal	Pros Better control over morphology, suitable for complex structures Cons Expensive solvents, longer reaction times	Energy conversion	Ramakrishnan et al. (2020)
Rutile, Anatase	Chemical vapor deposition (CVD)	Pros High purity, excellent control over film thickness and composition Cons Expensive equipment, high energy consumption, limited scalability	Thin films, electronic devices	Zeng et al. (2025)
Anatase, Rutile	Microwave Assisted	Pros Fast reaction times, energy-efficient, uniform heating Cons Limited to small-scale synthesis, requires specialized equipment	Photocatalysis	Ahmad et al. (2024)
Anatase	Green biosynthesis	Pros Environmentally friendly	Biomedical application	Nga and Alahmadi (2024), Sagadevan et al. (2022)

Several studies to synthesize TiO_2_NP using biomolecules from medicinal plants have been explored. For example, *Lippia citriodora* leaf extract has been used for the green synthesis of TiO_2_NPs, but its potential neurotoxicity requires further investigation (Campea et al., 2021). Plant extracts facilitate the green synthesis of TiO_2_NP through a multifaceted mechanism involving natural reducing and stabilizing agents (Sun et al., 2019). Phytochemicals such as polyphenols, flavonoids, and sugars donate electrons to titanium precursors like titanium tetraisopropoxide (TTIP) or titanium chloride (TiCl_4_), reducing the titanium ions (Ti^4+^) to TiO_2_NP (Sunny et al., 2022). This reduction is crucial for nanoparticle formation, as it initiates the nucleation and growth of TiO_2_. Concurrently, these phytochemicals act as stabilizers by adsorbing onto the nanoparticle surfaces, preventing aggregation through steric or electrostatic repulsion (Eddy et al., 2024). Complexation occurs when certain phytochemicals bind with titanium ions, controlling the reduction rate and influencing nanoparticle size and morphology (Timoszyk and Grochowalska, 2022). The plant-derived capping agents also modify the surface properties of the nanoparticles, enhancing their stability and functionality (Holghoomi and Colagar, 2024). This process not only reduces the reliance on toxic chemicals and harsh conditions typically used in conventional synthesis methods but also yields nanoparticles with potentially improved biocompatibility and environmental safety. By leveraging the natural properties of plant extracts, the green synthesis method provides a sustainable alternative that minimizes environmental impact and waste, making it especially suitable for biomedical applications.

In this work, we explore the use of *Morus alba* plant extract, rich in flavonoids with reported antioxidant, antimicrobial, anti-diabetic, anti-hyperlipidemic, anti-atherosclerotic, and anti-obesity properties (Pirsaheb et al., 2024), for the green synthesis of TiO_2_NP. This extract is expected to act as a reducing and complexing agent, leading to the formation of rod-shaped TiO_2_NP. This study highlights the potential of green synthesis methods utilizing plant extracts to produce TiO_2_NP for biomedical applications. The novelty of this study lies in the combination of eco-friendly synthesis methods and the integration of TiO_2_NP into a biocompatible hydrogel matrix. Unlike previous research that primarily focused on synthesizing TiO_2_NPs using plant extracts for various applications (Hussain et al., 2017a), this study emphasizes the direct biomedical application, particularly wound healing, by integrating these nanoparticles into a GG hydrogel. The research gap addressed here involves the challenge of creating a sustainable, biocompatible, and effective wound dressing material. Previous studies often stopped at the synthesis and characterization of TiO_2_NP using plant extracts (Sukidpaneenid et al., 2024), without exploring their incorporation into a functional hydrogel system designed for direct therapeutic use. This study not only advances the green synthesis of TiO_2_NP using plant extracts but also innovatively combines these nanoparticles with GG, which is known for its excellent film-forming ability, water retention, and biocompatibility (Abdl Aali and Al-Sahlany, 2024). This combination is specifically tailored for wound healing applications, aiming to harness the antimicrobial properties of TiO_2_NP along with the structural benefits of GG to create a novel wound dressing material. Therefore, the key novelty is the transition from mere nanoparticle synthesis to the development of a functional, green, and biocompatible wound dressing that leverages the benefits of both TiO_2_NP and GG, addressing both the environmental concerns of nanoparticle synthesis and the clinical need for effective wound care solutions.

## Experimental

### Materials

*Morus alba* leaves were collected from Kuala Nerus, Terengganu, Malaysia. Analytical grade titanium tetrachloride (TiCl_4_), ethanol and glycerol were obtained from Sigma Aldrich, Malaysia. Low-acyl gellan gum (Kelcogel) was purchased from a Huber Company, USA. Calcium chloride, and sodium hydroxide were obtained from Merck, Malaysia. Hydrochloric acid (37% concentrated) was acquired from HmbG. All materials were used as received.

### Preparation of aqueous leaf extract of Morus alba

Freshly harvested leaves of Morus alba underwent a rigorous decontamination process through repeated washing with distilled water and subsequent drying in a sterile environment for a period of 10 days. Subsequently, the desiccated Morus alba leaves were pulverized and sifted to obtain a fine powder. This Morus alba powder was then combined with ethanol in a ratio of 5 g–100 mL, and subjected to heating under reflux conditions at 50°C for a duration of 5 h to eliminate any pathogens present in the aqueous leaf extract solution. Ethyl alcohol served as the extraction medium in this process. Following this, the solution underwent filtration using Whatman No. 1 filter paper. The resulting filtrate was utilized in the production of titanium dioxide nanoparticles (TiO_2_NP).

### Preparation of titanium dioxide nanoparticle

TiO_2_NP were synthesized by adding 10 mL of filtered aqueous leaf extract solution to 100 mL of 5 mM TiCl_4_ (pH 1.5) in an Erlenmeyer flask under stirring at 50°C. After 5 h, the developed dark brown colour confirmed the formation of TiO_2_NP (Abdelmigid et al., 2022). Finally, well-formed TiO_2_NP were acquired by centrifugation at 10,000 rpm for 15 min and thus separated TiO_2_NP were dried and used for further analytical techniques.

### Preparation of titanium dioxide nanoparticles incorporated gellan gum hydrogel

Gellan gum (GG) solutions were prepared by dissolving 1 g GG in 100 mL deionized water under continuous stirring for 2 h at 70°C. To this solution, glycerol (5 wt%, relative to GG) and 5 mL CaCl_2_ (5 mM) were subsequently added. Following this, 0.01 g of biosynthesized TiO_2_NP was added to the solution while stirring for an additional 2 hours and sonication with 24 kHz Hielscher UP200H ultrasound (Hielscher Ultrasonics, Germany) for 5 min at ambient temperature. The resulting solution was then transferred to a casting dish, where it was dried in an oven for a period of 24 h at a temperature of 50°C, thus producing a 1wt%TiO_2_NP@GG hydrogel. Pure GG hydrogel was prepared suing similar procedure with absence of TiO_2_NP. The GG and TiO_2_NP@GG hydrogels were kept in refrigerator at 4°C, to maintain moisture and prevent microbial contamination, while avoiding freezing, which can affect the hydrogel’s integrity. Under these conditions, the hydrogel is stable and typically can be stored for 1–3 months. The hydrogels were stored in sealed, sterile, and dark containers to prevent light-induced degradation from TiO_2_’s photocatalytic properties.

### Characterization

X-ray diffraction patterns of TiO_2_NP, GG, and the TiO_2_NP@GG hydrogel were obtained utilizing a Rigaku Miniflex (II) X-ray diffractometer, with a scanning speed of 2.00° min^-1^. The diffraction patterns were captured within a range of diffraction angles (2θ) from 5° to 80° under ambient conditions. The morphology of the prepared specimens was examined employing a JOEL JSM 6360 LA scanning electron microscope, coated with Auto Fine Coats (JFC-1600) prior to analysis. XPS measurement was carried out using XPS Ultra Axis PLD, Kratos, employing Al (mono) Kα radiation (BE = 1486.7 eV). The broad survey scan was performed in the energy ranging of 0–1,000 eV. The binding energy (BE) for the samples was calibrated by setting the BE of C *1s* to 284.6 eV. The core level spectra of Ti *2p* and O *1s* were recorded through high resolution scans. The XPS spectra were further deconvoluted using *PeakFit program (version 4.12)*. For sample preparation, the hydrogel was simply loaded on the stub with double-sided adhesive tape was used to hold up the sample onto the stub. The stub then was placed in sample holder and transferred into machine chamber for measurement.

The mechanical characteristics of the TiO_2_NP@GG hydrogel and pure GG hydrogel were evaluated utilizing an Instron Universal Testing machine (model 3366) equipped with grips capable of bearing ±10 kN load and operating at a cross-speed of 10 mm min^-1^, adhering to ASTM D882 guidelines. Each specimen was precisely cut to dimensions of 2.0 × 6.0 cm for stress-strain assessments. The toughness (T) parameter was determined by integrating the area under the stress-strain curve of individual samples. The Young’s modulus (YM) and tensile strength (TS) were derived from the slope of the linear segment of the stress-strain curve and the peak stress, respectively. Elongation-at-break (E) was measured, with a minimum of three independent trials conducted for each specimen. Water Vapor Transmission Rates (WVTR) were evaluated following a customized ASTM International Standard procedure (ASTM. Designation E96-95, 1995). The samples were mounted on the circular opening of permeation bottles, having dimensions of 1.5 cm in diameter, 5.0 cm in height, and an effective transfer area (A) of 1.33 cm^2^. Subsequently, each sample was placed in a humidity chamber set at 37°C and 50% ± 5% RH. WVTR was determined by monitoring the rate of mass change (m) in these water-filled permeation bottles at exposure times (∆t = 24 h), calculated using Equation 1:

$$\text{WVTR}=\left(m/A∆t\right)$$ (1)

represents the amount of water gained per unit time of transfer, and A is the area exposed to water transfer (m^2^). The evaluation of the swelling properties involved the assessment of the weight of the dried hydrogel (W_dry_) before it was immersed in 50 mL phosphate buffer solutions with a pH of 7 at a temperature of 37°C ± 0.5°C. Subsequent to a 24-h period, the hydrogel was taken out, delicately wiped with tissue to eliminate surface moisture, and then re-measured (W_wet_). The percentage of water uptake was determined through the utilization of Equation 2.

$$\text{Water uptake }\left(\%\right)=\left(W_{\text{wet}}\;–\;W_{\text{dry}}\right)\;/\;W_{\text{dry}}$$ (2)

Where, W_dry_ and W_wet_ denote the initial weight and final weight, respectively. A minimum of five independent measurements were conducted for each sample.

### Anti-bacterial studies

Gram-positive bacteria, such as *Staphylococcus aureus* alongside Gram-negative bacteria, *Escherichia coli* was employed for antibacterial assessment. The sterilization of Muller-Hinton (MH) agar, a conventional growth medium, was carried out using an autoclave set at 120°C for a duration of 15 min. Subsequently, the bacterial strains were cultivated on MH agar plates and subjected to aerobic incubation at 37°C for a period of 24 h. Optical density readings of bacterial suspensions were collected at 600 nm through a Spectrophotometer Biomerieux Densicheck Plus to ensure a standardized inoculum of 0.5. The distribution of the bacterial cultures was uniformly performed on sterile Petri plates with MH agar utilizing a sterile cotton swab. Following this, the TiO_2_NP@GG hydrogel samples and control were delicately administered to the agar surface, with the insertion of sample discs measuring 6 mm in diameter. The plates were permitted to desiccate before the initiation of the 24-h incubation period at 37°C in triplicates. Any discernible clear zones surrounding the sample discs post the 24-h 37°C incubation period was duly documented as an indication of hindrance in microbial growth. A similar approach was employed to assess the antibacterial properties of TiO_2_NP@GG hydrogels with varying TiO_2_NP concentrations and pure TiO_2_NP (0.1 g).

A Fenton reaction between H_2_O_2_ and Fe^2+^ was used to produce •OH in order to examine the ability of •OH elimination (Rong et al., 2023). First, the same volume of H_2_O_2_ (9 mM) and FeSO_4_ (9 mM) were combined with 250 μL hydrogel ethanol solution (200 mg/mL). Next, to monitor •OH, 250 μL of salicylic acid ethanol solution (9 mM) was added to the combination. The supernatant was collected at various time intervals of 0.5, 1.0, 1.5, 2.5, and 3.0 h, and the absorbance at 510 nm was measured. The •OH scavenging was calculated as follow in Equation 3:

$$\cdot \text{OH scavenging }\left(\%\right)=\left(A_{b}-A_{h}\right)/A_{b}\times 100\;\%$$ (3)

Where *A*_*b*_ and *A*_*h*_ represented the control group (without hydrogel) and the TiO_2_NP@GG hydrogel group, respectively.

### Biocompatibility and cell proliferation studies

The propagation of 3T3 murine fibroblast cells necessitated the utilization of Dulbecco’s Modified Eagle Medium (DMEM, ATCC, USA) supplemented with 10% (v/v) fetal bovine serum (FBS, ATCC, USA) and 1% (v/v) antimicrobial agent (penicillin/streptomycin, ATCC, USA) as the nurturing medium. The cells were sustained at 37°C in a humidified 5% CO2 environment and sub-cultured every 3 days until attaining 60%–80% confluence. Before seeding the hydrogel specimens, they underwent sterilization via UV irradiation for 30 min in a biosafety cabinet and subsequently positioned in a 96-well plate (Nunc, Germany). To conduct cell viability assessments, hydrogel specimens were immersed in DMEM culture medium for 24 h, followed by removal of the supernatant prior to seeding 3T3 murine fibroblast cells (5,000 cells/well) into the wells. The cells were then nurtured at 37°C in a humidified 5% CO_2_ environment for 24, 48, and 72 h. The DMEM culture medium lacking hydrogel specimens functioned as the control group. Assessment of cell viability was carried out after 24, 48, and 72 h of incubation through a staining technique involving acridine orange/propidium iodide (AO/PI, Sigma Aldrich, USA) and visualization under a light microscope (Olympus IX73-FL-CCD) equipped with a fluorescence filter (Olympus U-LH100HG with blue light excitation). Preceding observation, the media in each well were substituted with fresh media. The proliferation of 3T3 murine fibroblast cells was quantified utilizing MTT (3-(4,5-dimethylthiazol-2-yl)-2,5-diphenyltetrazolium bromide) (Thermo Fisher Scientific, USA). All wells containing hydrogel specimens and the control group were exposed to 50 μL of MTT assay solution and incubated for 4 h. Subsequently, the solution was substituted with dimethyl sulfoxide (DMSO, Merck, USA), and after 30 min, absorbance was gauged at 520 nm employing a microplate reader (Multiskan Ascent 96/384, USA) subsequent to agitation at 490 nm for 15 min. The absorbance values were translated into cell quantities utilizing calibration curves of 3T3 murine fibroblast cells in 96-well plates under identical conditions.

### *In vitro* wound healing studies

Wound healing experiments *in vitro* were carried out employing a scratch wound assay, as detailed in a previous publication (Doostan et al., 2023). The scratch wound assay represents a widely recognized technique for assessing the migratory potential of fibroblasts and their capacity to seal wounds, a critical aspect in the process of wound healing (You et al., 2017; Radstake et al., 2023). 3T3 fibroblast cells were cultured in a 24-well plate for 24 h to establish a cell monolayer with 80%–90% confluence. Following this, a serum-free media supplemented with mitomycin C (5 g/mL) was applied to the cells for 12 h to impede cell proliferation, succeeded by a thorough wash to eliminate any remaining mitomycin C. Linear wounds were then generated in the cell monolayers using a micropipette tip, resulting in areas devoid of cells. The scratched wells underwent three washes with PBS to get rid of any floating or detached cells. Each well was filled with medium containing sterile hydrogels and placed in an incubator at 37°C with 5% CO_2_. The untreated monolayer of scratched cells in the complete media was designated as the control. The closure rate of the wounds was observed and recorded 24 h post-scratching using an inverted microscope, with corresponding images captured. Subsequently, ImageJ software was employed for the analysis and quantification of the open wound area at the specified time.

## Results and discussion

The biosynthesis of TiO_2_ nanoparticles (TiO_2_NP) employed TiCl_4_ as the titanium precursor and phytochemicals from *M. alba* leaf extract as the reducing agent. These phytochemicals, rich in hydroxyl (•OH) groups, play a key role in the reduction process by donating electrons to metal ions (Hussain et al., 2017b; Ovais et al., 2018). Polyphenolic tannins within the extract likely complex with Ti^4+^ ions from the precursor, facilitating the formation of TiO_2_NP through an electron transfer mechanism (Goutam et al., 2018). Water-soluble heterocyclic compounds in the reaction medium are believed to contribute to nanoparticle stabilization (Reddy et al., 2019; Srinivasan et al., 2019). Equations 4, 5 depict the postulated reactions involved in the conversion of TiCl_4_ to TiO_2_NP.

$${\text{TiCl}}_{4}+{\text{xH}}_{2}O\;\to \text{ Ti}{\left(\text{OH}\right)}_{x}{\text{Cl}}_{4\;–\;x}+x\text{ HCl}$$ (4)

$$\text{Ti}{\left(\text{OH}\right)}_{x}{\text{Cl}}_{4\;–\;x}\;\to \;{\text{TiO}}_{2}+\left(4\;–\;x\right)\text{ HCl}+\left(x-2\right)\;H_{2}O$$ (5)

**FIGURE 1:**
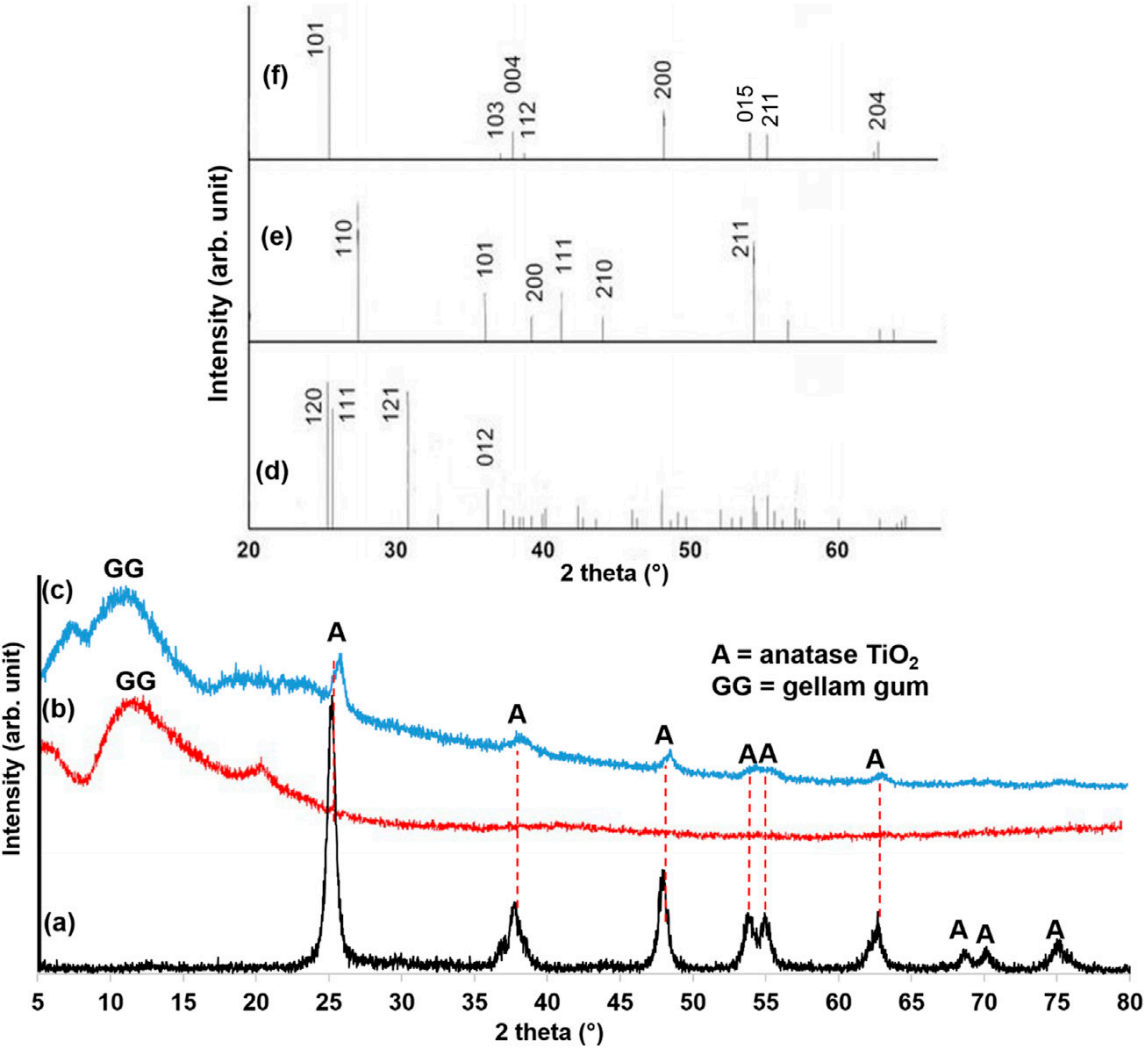
XRD patterns of **(a)** biosynthesis TiO_2_NP **(b)** GG hydrogel **(c)** TiO_2_NP@GG hydrogel **(d)** TiO_2_ brookite (JCPDS card no. 29–1360) **(e)** TiO_2_ rutile (JCPDS card no. 21–1276), and **(f)** TiO_2_ anatase (JCPDS card no. 21–1272).

The higher ligand field strength of hydroxide (OH^−^) ions compared to chloride (Cl^−^) ions promotes the displacement of Cl^−^ from TiCl_4_, leading to rapid hydrolysis in the presence of water. The resulting Ti(OH)_x_Cl_4-x_ undergoes polycondensation, forming an extensive Ti-O-Ti network characteristic of TiO_2_. X-ray diffraction (XRD) analysis confirmed the successful formation of anatase TiO_2_ (Figure 1a). The presence of distinct peaks at 25°, 38°, 48°, 54°, 55°, 63°, 69°, 70°, and 75° corresponds to the (101), (004), (200), (015), (211), (204), (116), (220), and (125) planes of anatase TiO_2_, respectively (Akram et al., 2024). The peaks also match with the standard peak positions for anatase phases of TiO_2_ from the Joint Committee on Powder Diffraction Standards (JCPDS), card no. 21–1272 (Scarpelli et al., 2018). Conversely, the XRD pattern of the synthesized GG hydrogel (Figure 1b) exhibited a broad peak around 11°, characteristic of its amorphous nature (Zhou et al., 2024). The XRD pattern of the TiO_2_NP@GG hydrogel (Figure 1c) confirmed the successful loading of TiO_2_NP onto the GG matrix. A slight shift in the anatase TiO_2_ peaks suggests an interaction between the crystalline nanoparticles and the biopolymer matrix, potentially indicating the formation of a nanocomposite (Zhou et al., 2009). The standard peak positions of brookite, rutile, and anatase TiO_2_ are shown in Figures 1d-f, respectively.

**FIGURE 2:**
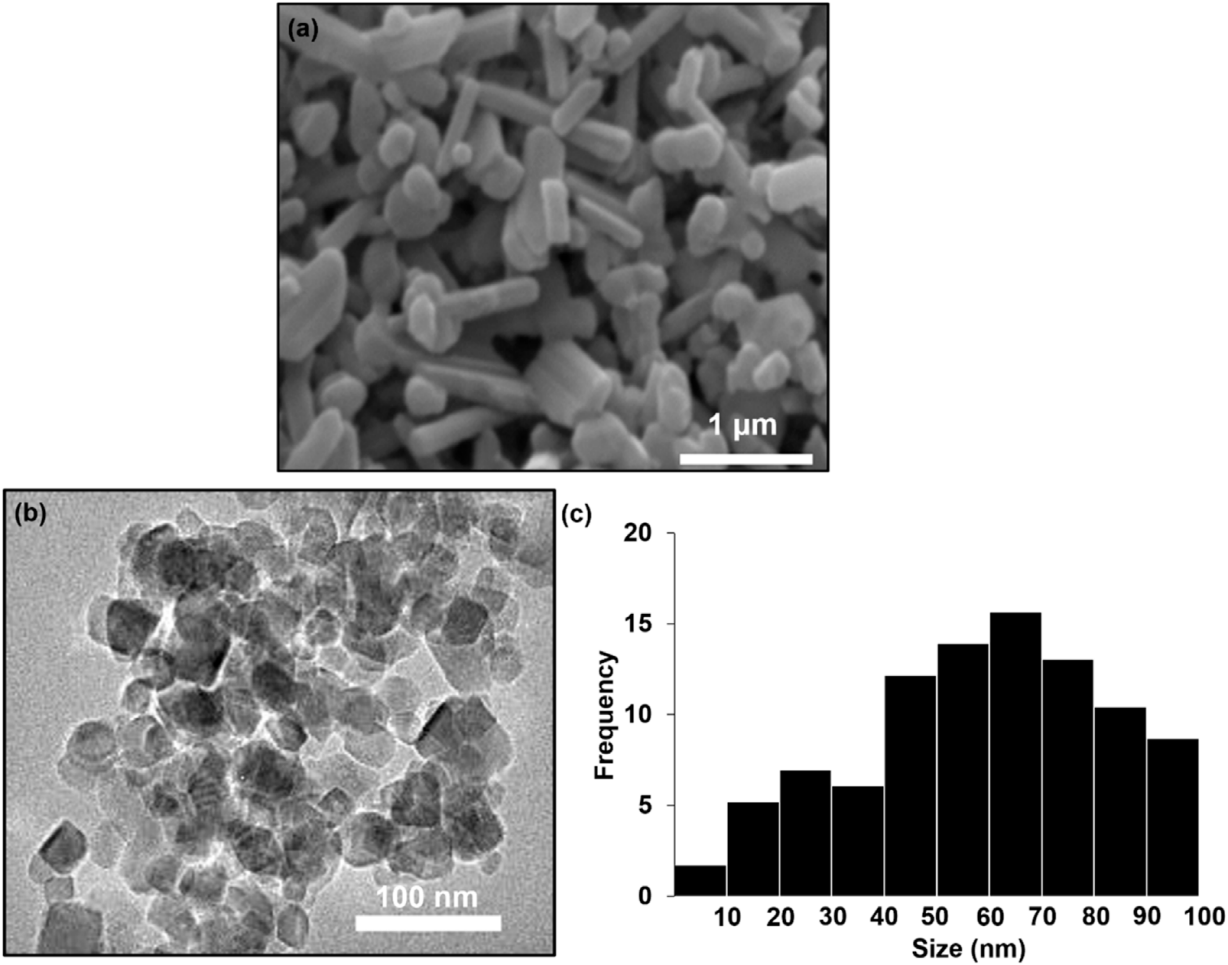
**(a)** SEM image **(b)** TEM image and **(c)** particles size distribution (PSD) biosynthesis TiO_2_NP.

Scanning electron microscopy (SEM) micrograph reveal the morphology of the biosynthesized TiO_2_NP. As shown in Figure 2a, the TiO_2_NPs exhibit an irregular, predominantly rod-like morphology, likely due to the aggregation of smaller anatase nanoparticles during the drying process. Notably, the particle size of the TiO_2_NP falls within the nanoscale range (80–90 nm), demonstrating the effectiveness of *Morus alba* extract in producing nanomaterials. The transmission electron microscopy (TEM) images in Figure 2b, show that the particles are in irregular shape and have sizes ranging from 60 to 90 nm, consistent with the SEM results. The particles size distribution (PSD) analysis revealed that the majority of the particles fall within the size range of 50–90 nm (Figure 2c. This confirms the nanoscale nature of the biosynthesized TiO_2_NP.

**FIGURE 3:**
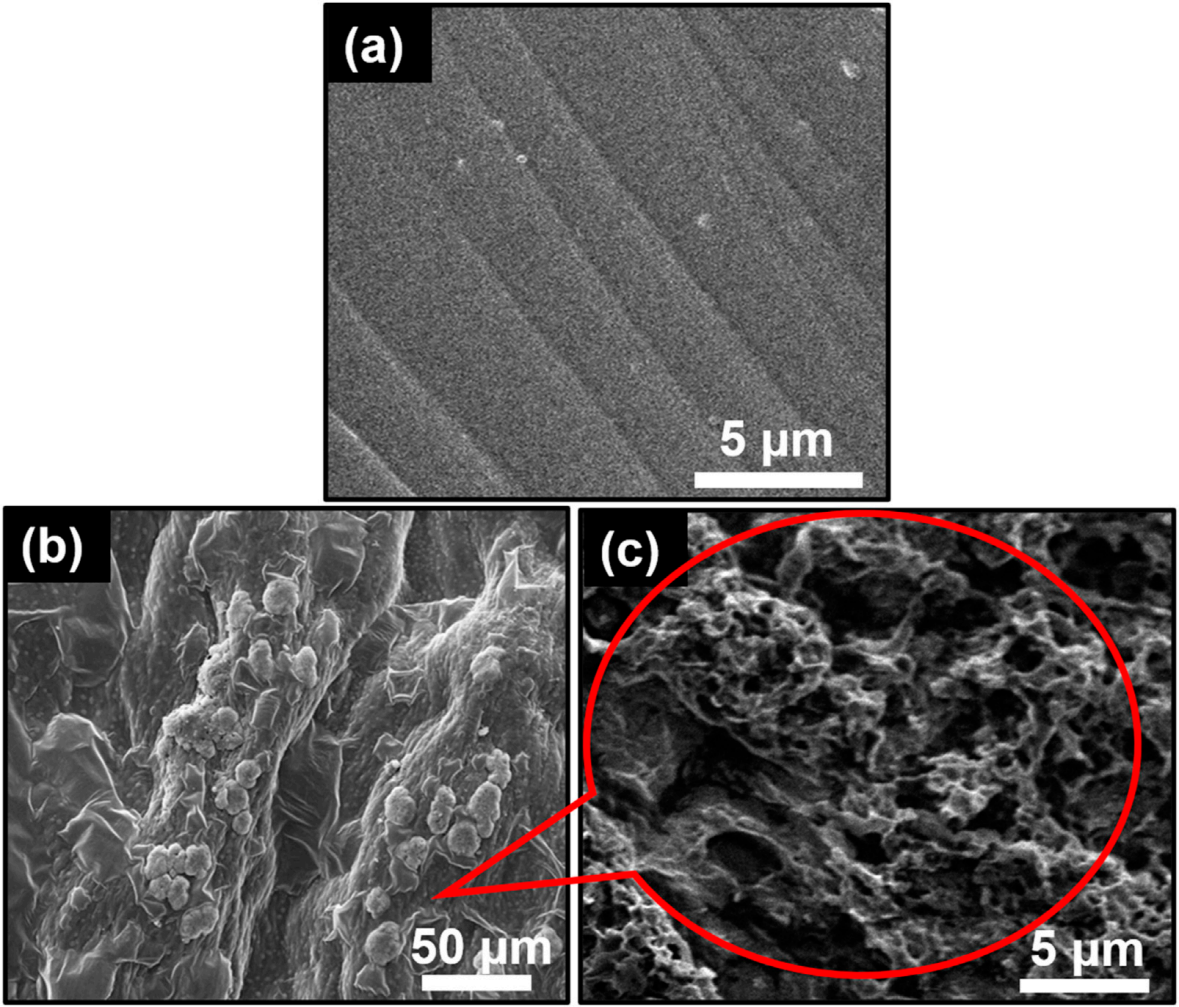
SEM images of **(a)** GG **(b)** TiO_2_NP@GG hydrogel and **(c)** TiO_2_NP@GG hydrogel at higher magnification.

The SEM image of pure GG hydrogel displays a uniform surface with slight protuberances (Figure 3a, indicating homogeneous blending of the GG matrix. The low concentration of GG likely contributes to a uniform hydrogel structure due to strong bonds between hydrophilic components (Alizadeh-Sani et al., 2020). In contrast, the SEM image of the TiO_2_NP@GG hydrogel (Figure 3b reveals a heterogeneous surface with agglomerated TiO_2_NP attached to the surface of the GG matrix. This surface attachment is likely due to the migration of TiO_2_NP during the drying process, which can result in a non-uniform distribution of nanoparticles within the hydrogel (Mohamed et al., 2025). The agglomeration is a common phenomenon attributed to minimization of surface energy. The interaction between Ti-O groups on the TiO_2_NP and COO–groups on the GG backbone can lead to interpolymer complex layer formation, resulting in a roughened hydrogel surface (Crosby and Lee, 2007). A higher magnification SEM image of the TiO_2_NP@GG hydrogel (Figure 3c further confirms the presence of these agglomerated TiO_2_NP.

**FIGURE 4:**
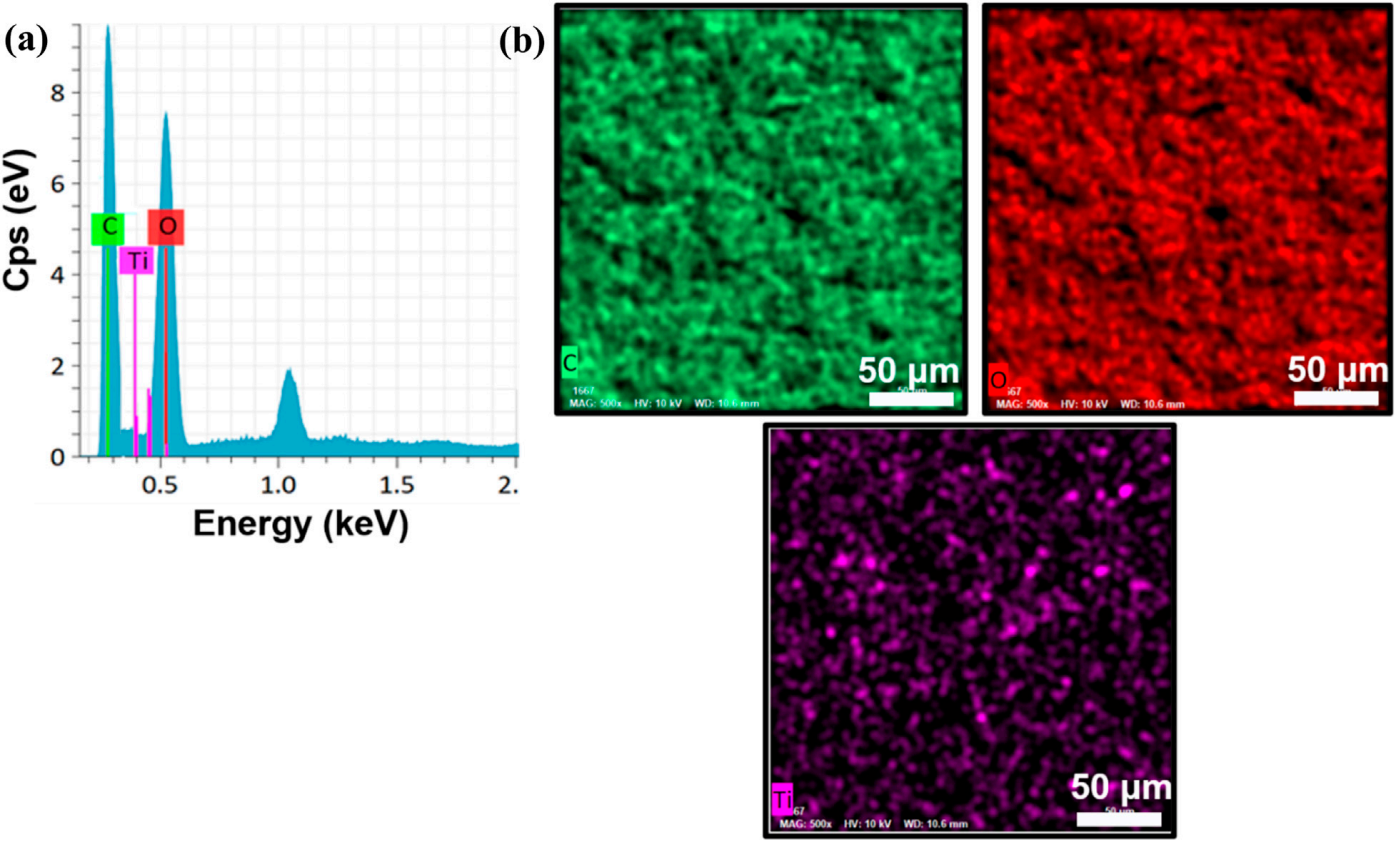
**(a)** EDS spectra and **(b)** elemental mapping of the synthesized TiO_2_NP@GG hydrogel.

Energy-dispersive X-ray spectroscopy (EDAX) analysis (Figure 4a confirms the presence of titanium (Ti), oxygen (O), and carbon (C) elements within the TiO_2_NP@GG hydrogel with the composition of 0.66 wt%, 52.54 wt%, and 46.80 wt% respectively. The peak observed at 1.0 keV in the EDAX spectrum is attributed to the presence of sodium (Na), which is likely a residual element from the synthesis process or the GG matrix. Na is a common contaminant in EDAX analysis and is often detected at low energies. Additionally, Figure 4b demonstrates a good distribution of these elements throughout the hydrogel matrix. To further validate the findings, the X-ray photoelectron spectroscopy (XPS) analysis was conducted to complement the EDAX results.

**FIGURE 5:**
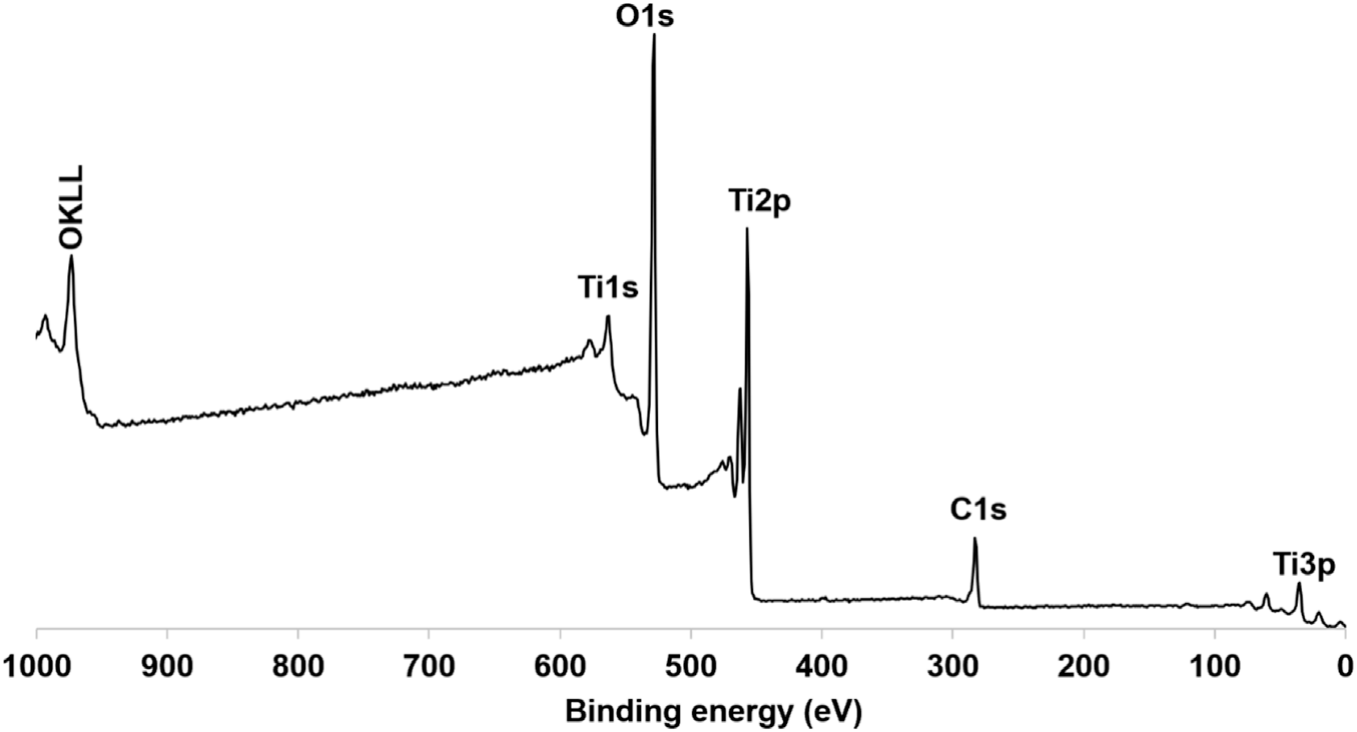
XPS spectra of synthesis TiO_2_NP@GG hydrogel.

XPS analysis was employed to further investigate the elemental composition and chemical oxidation states within the TiO_2_NP@GG hydrogel (Figure 5). The presence of titanium (Ti), oxygen (O), and carbon (C) corresponding to the expected elements in the sample, confirming the presence of TiO_2_ and the organic components of the GG matrix.

**FIGURE 6:**
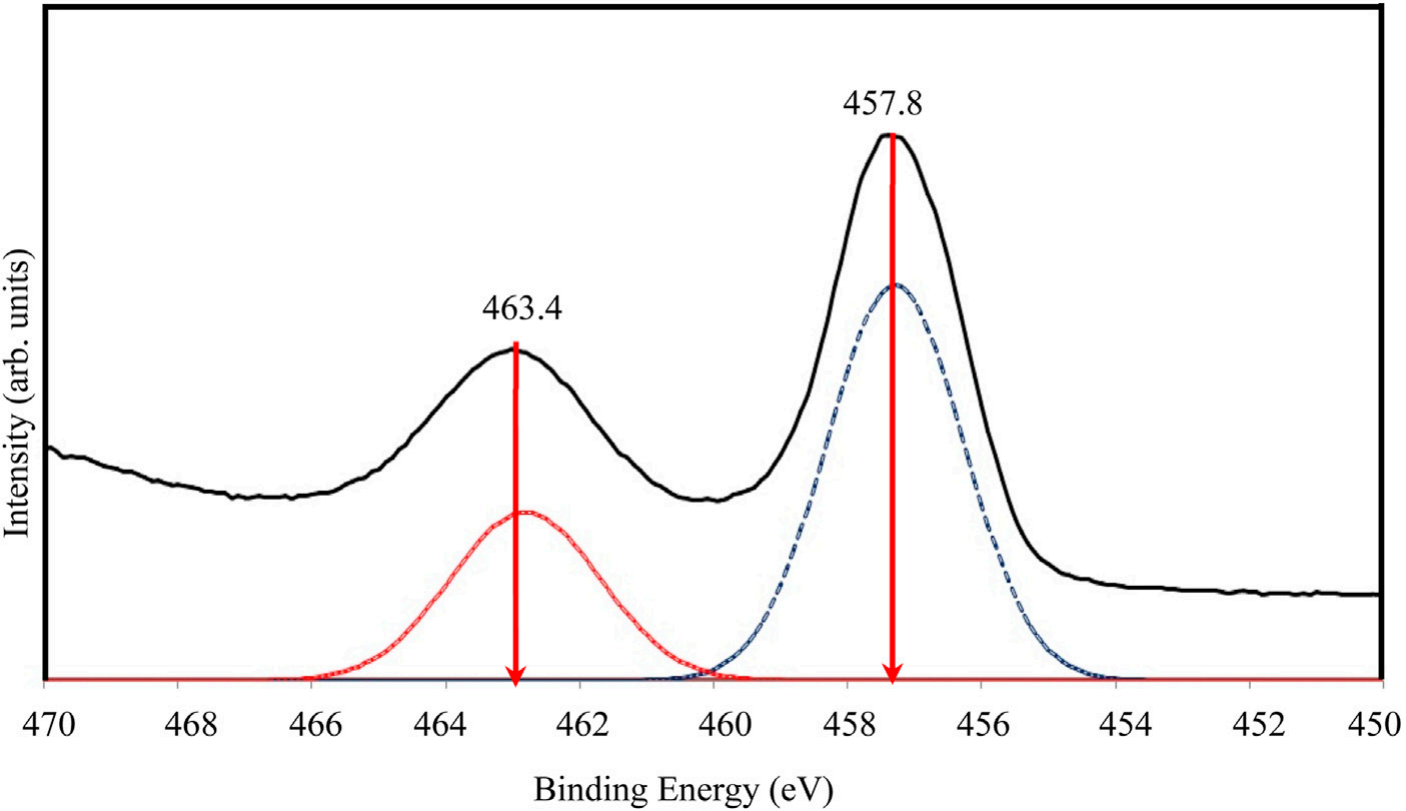
High resolution of XPS spectra for Ti 2p of TiO_2_NP@GG hydrogel; XPS analysis peak (solid line) and deconvolution peak (doted line).

Figure 6 presents the high-resolution XPS spectra for Ti 2p. Due to the broad peak, a deconvolution process was performed using PeakFit software based on the Gaussian fit principle. Deconvolution revealed two distinct peaks at 463.4 eV and 457.8 eV, corresponding to Ti-O bonding in TiO_2_ and attributed to Ti^4+^ 2p_1/2_ and Ti^4+^ 2p_3/2_ spin-orbital splitting photoelectrons, respectively. This confirms the presence of Ti^4+^ in the sample (Yang et al., 2024).

**FIGURE 7:**
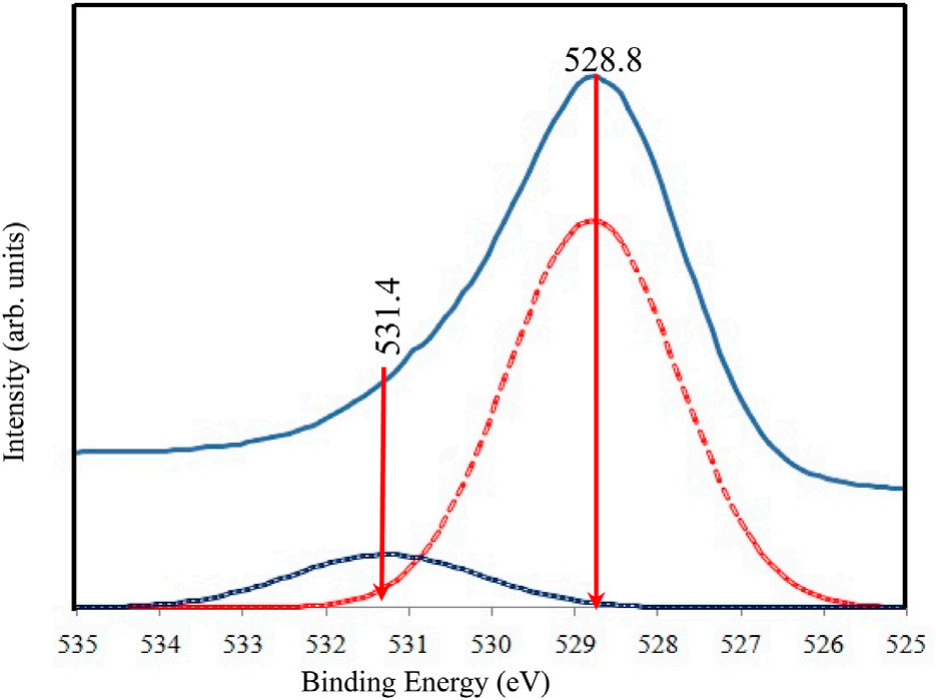
High resolution of XPS spectra for O 1s of TiO_2_NP@GG hydrogel; XPS analysis peak (solid line) and deconvolution peak (doted line).

The high-resolution XPS spectra for O 1s are shown in Figure 7. The broad and asymmetric peak suggests the presence of multiple oxygen chemical states. Following deconvolution, two peaks were identified in the O 1s region. The primary peak at approximately 528.8 eV aligns with oxygen bound to Ti^4+^ in TiO_2_ (O-Ti-O), consistent with the binding energy of O^2-^ in the TiO_2_ lattice (Qahtan et al., 2024). A secondary shoulder peak at a higher binding energy (531.4 eV) is also observed and is attributed to adsorbed oxygen, potentially due to the presence of water molecules within the sample.

Table 2 summarizes the mechanical properties (tensile strength (TS), Young’s modulus (YM), toughness (T), and elongation-at-break (EAB)) of pristine GG and GG@TiO_2_NP hydrogels. Compared to pure GG, the GG@TiO_2_NP hydrogel exhibited enhanced mechanical strength, with TS and YM increasing from 1.42 ± 0.12 MPa to 4.48 ± 0.11 MPa and 20.22 ± 2.11 MPa to 77.48 ± 2.24 MPa, respectively. The incorporation of rod-like TiO_2_NPs likely facilitated efficient stress transfer between the filler and biopolymer chains due to strong surface interactions, leading to a significant increase in hydrogel strength. This improved strength could be beneficial for promoting fibroblast cell proliferation, making it potentially valuable for wound healing applications (Behera et al., 2017). The role of rod-like nanoparticles in enhancing the mechanical properties of polymer composites is well-documented in the literature. For instance, studies have shown that rod-like or anisotropic nanoparticles, such as TiO_2_ nanorods, exhibit strong interfacial interactions with polymer matrices due to their high surface area and aspect ratio (Abdal-hay et al., 2014). These interactions facilitate efficient stress transfer from the polymer matrix to the nanoparticles, leading to improved mechanical strength (Cazan et al., 2021; Arya et al., 2019; Sokhandani et al., 2013). In this study, the observed increase in hydrogel strength is consistent with these findings and supports the proposed mechanism of stress transfer.

**TABLE 2:** Mechanical properties, WVTR and swelling of GG and TiO_2_-NP@GG hydrogel.

Sample	TS (MPa)	YM (MPa)	T (J g^−1^)	EAB (%)	WVTR (g m^−2^ d^−1^)	Swelling (%)
GG	1.42 ± 0.12	20.22 ± 2.11	0.18 ± 0.08	11.24 ± 0.58	558 ± 3	1002 ± 8
TiO_2_NPs@GG	4.48 ± 0.11	77.48 ± 2.24	0.16 ± 0.02	9.91 ± 0.65	485 ± 4	1482 ± 6

Mean ± SD, n = 5; TS, tensile strength; YM, Young’ Modulus; T, toughness; EAB, elongation-at-break; WVTR, water vapor transmission rate.

However, T and EAB decreased slightly (approximately 10% each) upon the introduction of TiO_2_NPs. This decrease can be attributed to the uniform dispersion of TiO_2_NPs via interfacial interactions like electrostatic forces, hydrogen bonding, and O-Ti-O bonds with other hydrogel components. These interactions enhance cohesive forces within the sample, restricting the movement of the polymer network (He et al., 2016). Additionally, the incorporation of TiO_2_ into the biopolymer matrix increases its rigidity (Jagadeesh et al., 2021), resulting in lower EAB values.

Table 2 also presents the water vapor transmission rate (WVTR) and swelling behavior of the fabricated hydrogels. The TiO_2_NP@GG hydrogel displayed a marginally lower WVTR compared to the pure GG hydrogel. This can be explained by the nanoparticles acting as fillers within the biopolymer matrix, occupying voids and interstices. Additionally, the nanoparticles interact with the polymer matrix, forming stable hydrogen bonds. These gaps within the macromolecular structure typically accommodate water molecules. However, the introduction of nanoparticles fills these vacancies, enhancing structural rigidity and consequently reducing water vapor permeation (Ghazihoseini et al., 2015). The aggregation of nanoparticles on the hydrogel surface can also modify the stiffness and porosity of the matrix, further hindering water ingress (Abdullah et al., 2019). Similar observations have been reported in previous studies where WVTR decreased with the addition of nanoparticles to biopolymer matrices (Saedi et al., 2020; Tabatabaei et al., 2018).

Interestingly, the TiO_2_NP@GG hydrogel exhibited significantly higher swelling (1482% ± 6%) compared to pure GG (1002% ± 8%). The integration of TiO_2_NP increases the hydrogel’s surface area-to-volume ratio, facilitating enhanced water absorption by providing more space for water molecules to readily adsorb within the hydrogel network. Furthermore, the hydrophilic nature of the TiO_2_NP material also contributes to the increased swelling propensity of the TiO_2_NP@ GG hydrogel (Liu et al., 2024).

**FIGURE 8:**
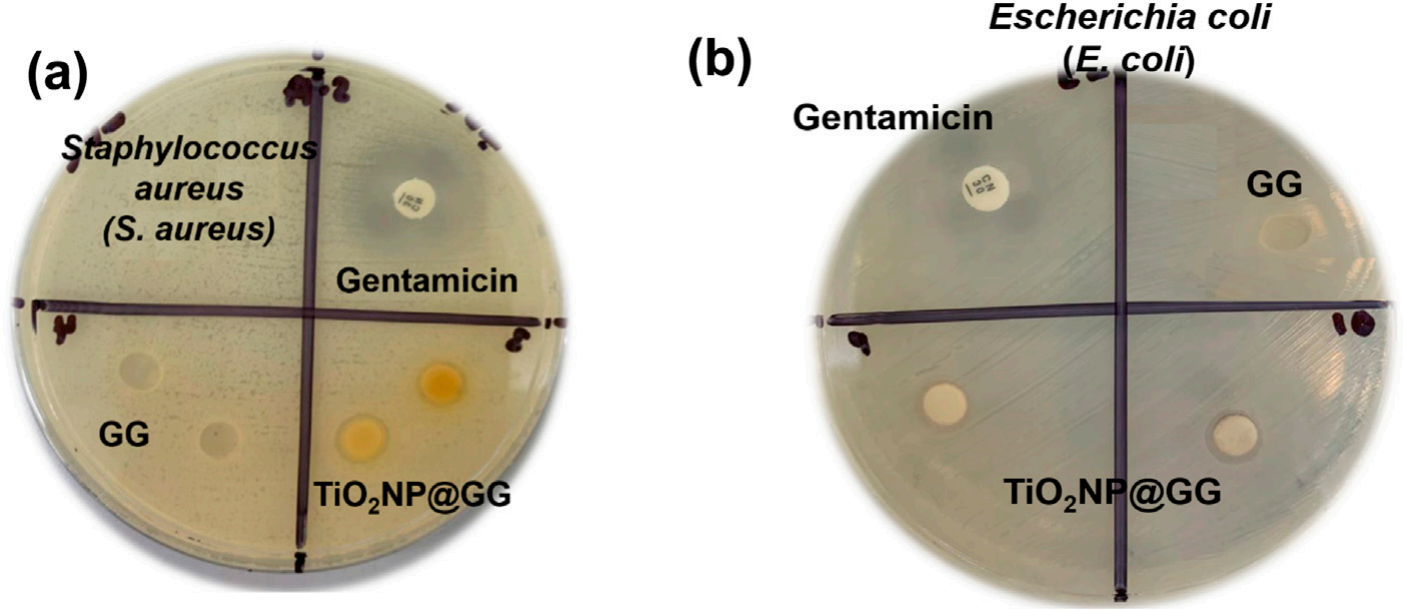
Disk diffusion antibacterial activity of gentamicin (positive control), GG and TiO_2_NP@GG hydrogel against the **(a)** *Staphylococcus aureus* (*S. aureus)* **(b)** *Escherichia coli (E. coli)* after incubating for 24 h.

The disc diffusion method was employed to evaluate the antibacterial activity of pure GG and TiO_2_NP@GG hydrogels against Gram-positive *Staphylococcus aureus* (*S. aureus*) and Gram-negative *Escherichia coli* (*E. coli*) bacteria. After 24 h of incubation, no inhibition zone was observed for the pure GG hydrogel against *S. aureus* (Figures 8a and *E. coli* Figure 8b). Conversely, the TiO_2_NP@GG hydrogel exhibited a clear inhibition zone around the sample, measuring 5.06 ± 0.04 mm and 4.22 ± 0.06 against the *S. aureus and E. coli*, respectively comparable to the gentamicin as control (Table 3).

**TABLE 3:** Quantitative results of gentamicin, GG and TiO_2_NP@GG hydrogels against the *Staphylococcus aureus* and *Escherichia coli* bacteria as indicated by the zone of inhibition (mm).

Inhibition zone (mm)		
Material	*S. aureus*	*E. coli*
Gentamicin	15.01 ± 0.02	13.08 ± 0.04
GG	—	—
TiO_2_NP@GG	5.06 ± 0.04	4.22 ± 0.06

Data are presented as mean ± standard deviation (n = 3).

**FIGURE 9:**
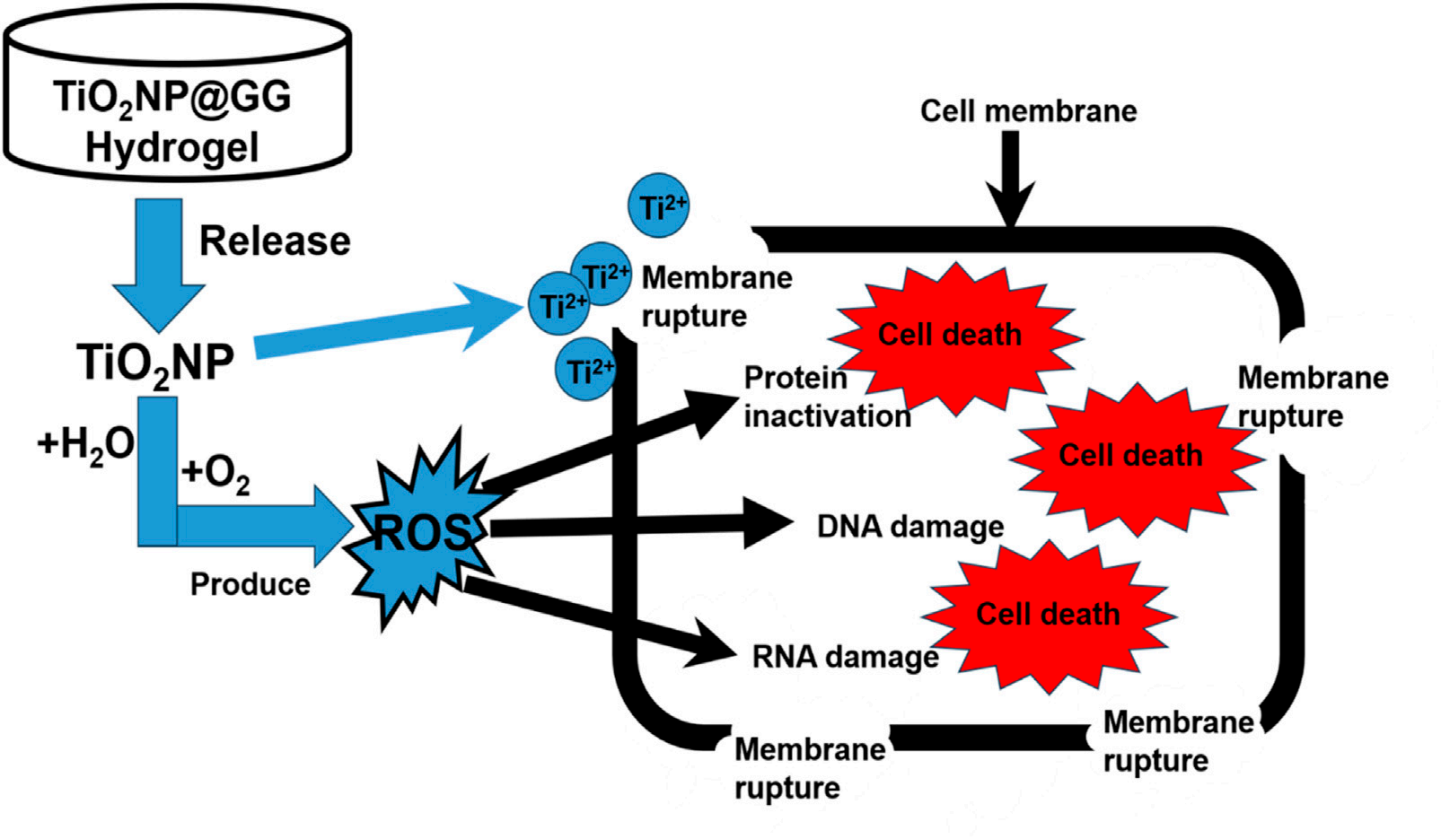
Proposed antibacterial mechanisms of TiO_2_NP@GG hydrogel via ROS generation.

The antimicrobial activity of the TiO_2_NP@GG hydrogel can be attributed to interactions between the nanoparticles and bacterial components. Upon contact with the treated material surface, a potential electrostatic interaction occurs between the negatively charged bacterial membranes and the positively charged metal oxide nanoparticles (Shoudho et al., 2024; Metryka et al., 2024; Issler et al., 2024). This interaction can lead to the generation of reactive oxygen species (ROS), causing oxidative stress and ultimately cell death (Rauf et al., 2024). Figure 9 shows the proposed antibacterial mechanisms of TiO_2_NP@GG hydrogel via ROS generation.

ROS including superoxide anion, hydroxyl anion, hydrogen peroxide, and hydroxyl free radicals (•OH), are commonly present with •OH being recognized as one of the most reactive species (Kozlov et al., 2024; Alanazi et al., 2024). In this study, we evaluated the •OH scavenging capabilities of GG and TiO_2_NP@GG hydrogels. The Fenton reaction was used to generate •OH, while salicylic acid was employed as an indicator. The Fenton reaction is a well-established process that involves the reaction of hydrogen peroxide (H_2_O_2_) with ferrous ions (Fe^2+^) to generate hydroxyl radicals (•OH) as in Equation 5 (Sasak et al., 2024);

$${\text{Fe}}^{2+}+H_{2}O_{2}\;\to \;{\text{Fe}}^{3+}+\cdot \text{OH}+{\text{OH}}^{-}$$

**FIGURE 10:**
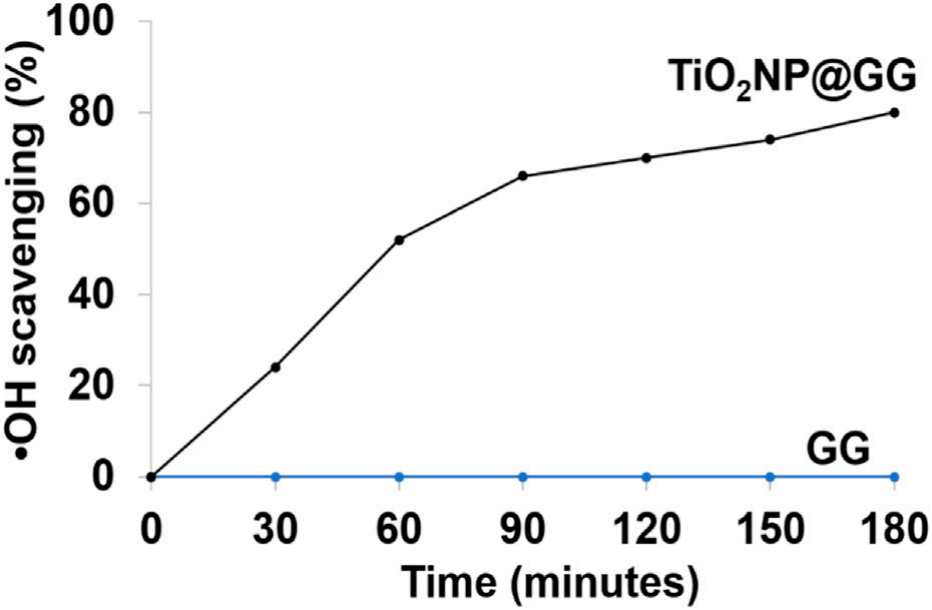
Scavenging results of hydroxyl free radicals (•OH) using pure GG and TiO_2_NP@GG hydrogels.

As shown in Figure 10, the pure GG hydrogel displayed no scavenging activity. However, the TiO_2_NP@GG hydrogel exhibited an increase in •OH scavenging over time and approximately 80% of •OH scavenging within 3 h, effectively mitigating oxidative stress.

Additionally, nanoparticles may interact with phosphorus or sulfur-based compounds within bacteria, such as DNA and protein thiol groups. This disrupts vital cellular functions by hindering DNA replication and deactivating proteins, leading to increased cell permeability and cell death (Khalandi et al., 2017). Recent studies suggest that metal nanoparticles might establish strong electrostatic bonds with bacterial cell membranes, further enhancing their antibacterial efficacy against various microbial pathogens (Shaikh et al., 2019; Franco et al., 2022; Salas-Orozco et al., 2024).

The antibacterial efficacy of TiO_2_NP@GG hydrogels, containing 0.1, 0.5, 1.0, and 5.0 weight percent (wt%) of TiO_2_NP, along with pure TiO_2_NP, was evaluated against *S. aureus* and *E. coli*. The results are summarized in Table 4. The hydrogel with 0.1wt% TiO_2_NP exhibited inhibition zones of 3.10 ± 0.02 mm and 2.42 ± 0.02 mm against *S. aureus* and *E. coli*, respectively. As the TiO_2_NP content increased to 0.5, 1.0, and 5.0wt%, the inhibition zones expanded to 4.41 ± 0.04 mm, 5.06 ± 0.04 mm, and 7.02 ± 0.10 mm against *S. aureus*. Similarly, against *E. coli*, the inhibition zones increased to 2.88 ± 0.04 mm, 4.22 ± 0.06 mm, and 6.48 ± 0.11 mm. Notably, the 5wt% TiO_2_NP@GG hydrogel demonstrated the largest inhibition zones.

**TABLE 4:** Inhibition zone of TiO_2_NP@GG hydrogel at different weight percent of TiO_2_NP in hydrogel

Sample		Inhibition zone (mm)	
	TiO_2_NP content (g)	*S. aureus*	E. coli
0.1wt%TiO_2_NP@GG	0.001	3.10 ± 0.02	2.42 ± 0.02
0.5wt%TiO_2_NP@GG	0.005	4.41 ± 0.04	2.88 ± 0.04
1wt%TiO_2_NP@GG	0.010	5.06 ± 0.04	4.22 ± 0.06
5wt%TiO_2_NP@GG	0.050	7.02 ± 0.10	6.48 ± 0.11
TiO_2_NP	0.100	8.01 ± 0.12	6.88 ± 0.14

Data are presented as mean ± standard deviation (n = 3).

The enhancement in antibacterial activity of TiO_2_NP@GG hydrogel can be attributed to the increased generation of reactive oxygen species (ROS) with higher TiO_2_NP concentrations, which are known to enhance antimicrobial effects (Hao et al., 2024). The antibacterial activity of TiO_2_NP@GG hydrogels is attributed to the generation of reactive oxygen species (ROS) at the surface of TiO_2_NPs. Under light irradiation, TiO_2_NPs undergo photoexcitation, producing electrons (e^−^) and holes (h^+^) that react with adsorbed oxygen (O_2_) and water (H_2_O) to form ROS, such as superoxide radicals (•O_2_^−^) and hydroxyl radicals (•OH) (Badoni et al., 2025). These ROS cause oxidative damage to bacterial cell membranes, proteins, and DNA, leading to cell death (Nikolaou et al., 2025).

The comparable inhibition zones observed for pure TiO_2_NPs (8.01 ± 0.12 mm) and TiO_2_NP@GG hydrogels confirm that the antibacterial effect is primarily due to the ROS generated at the surface of TiO_2_NPs, even when embedded in the GG matrix. Despite the lower TiO_2_NP concentration in the hydrogel the inhibition zone radius of 5wt%TiO_2_NP@GG closely resembles that of bare TiO_2_NP. This suggests that the GG matrix effectively retains and delivers TiO2NPs to the bacterial cells, maintaining their antibacterial efficacy. However, the inhibition zones of both TiO_2_NP@GG hydrogel and bare TiO_2_NPs are less than 50% of the control (Gentamicin), which can be attributed to differences in their mechanisms of action, concentration, and delivery efficiency. Gentamicin directly inhibits bacterial protein synthesis, leading to rapid cell death, while the antibacterial activity of TiO_2_NP is mediated by ROS generation, which is slower and less direct. Additionally, the GG matrix may slow down the diffusion of TiO_2_NP, further contributing to the observed differences in antibacterial activity.

Notably, excessive TiO_2_NP concentrations can lead to nanoparticle aggregation, which reduces the available surface area and may diminish the antibacterial activity, as noted by other researchers (Wang et al., 2024; Caselli et al., 2024; Baamer et al., 2024).

**FIGURE 11:**
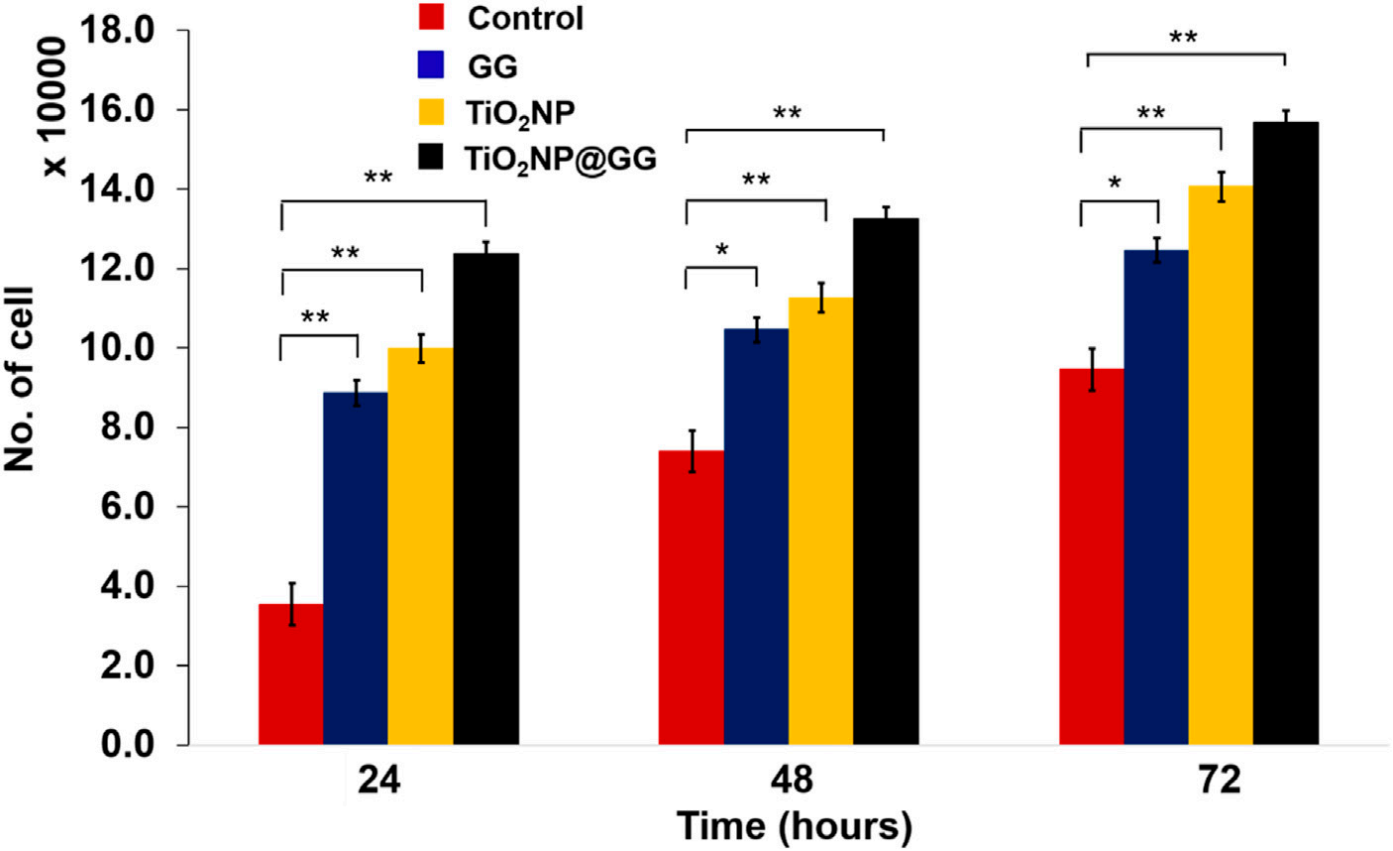
Cell proliferation for the TCPP control sample, GG, bare TiO_2_NP and TiO_2_NP@GG hydrogel cultured in the medium containing 3T3 mouse fibroblast cells. Data are presented as mean ± standard deviation (n = 3) and error bars indicated as standard deviation. Statistically significant differences compared to control are indicated by p < 0.05 (*) and p < 0.01 (**).

The cytocompatibility of the hydrogel samples was assessed *in vitro* using 3T3 mouse fibroblast cells (Figure 11). All samples exhibited an increase in cell number from 24 to 72 h, indicating their cytocompatibility and non-toxic nature. Notably, the TiO_2_NP@GG hydrogel displayed the highest cell proliferation compared to the pure GG hydrogel, bare TiO_2_NP and tissue culture polystyrene plate (TCPP) control. TCPP were used as the control in cell culture experiments. TCPP is composed of polystyrene with a chemically modified surface to enhance cell adhesion and proliferation. It is widely recognized as a standard control material in cell culture studies due to its biocompatibility, consistency, and ability to support cell growth. The use of TCPP as a control allowed us to evaluate the performance of TiO_2_NP@GG hydrogels relative to a well-characterized and widely accepted benchmark.

By 72 h, the cell count reached approximately ∼156,740 cells/well for the TiO2NP@GG hydrogel, compared to ∼140,520 cells/well for bare TiO_2_NP, ∼132,440 cells/well for the GG hydrogel, and ∼123,738 cells/well for the TCPP control. The higher cell proliferation observed with bare TiO_2_NP compared to the GG hydrogel and TCPP control suggests that TiO_2_NP alone promote cell growth, likely through enhanced protein interaction, cell adhesion, and proliferation, which aligns with observations from previous studies (Li et al., 2022). The highest cell proliferation observed with the TiO_2_NP@GG hydrogel indicates a synergistic effect between the TiO_2_NP and the GG matrix, further enhancing cell growth.

The efficacy of the hydrogels as wound dressings was evaluated using the scratch assay, a well-established *in vitro* wound healing model. The assay involves creating a scratch or gap in a confluent monolayer of cells and monitoring the migration of cells into the scratched area over time. This method mimics the process of wound healing and provides insights into the effects of materials on cell migration. The study evaluated four distinct sample groups which are TCPP as control sample, bare TiO_2_NP, GG hydrogel and TiO_2_NP@GG hydrogel.

**FIGURE 12:**
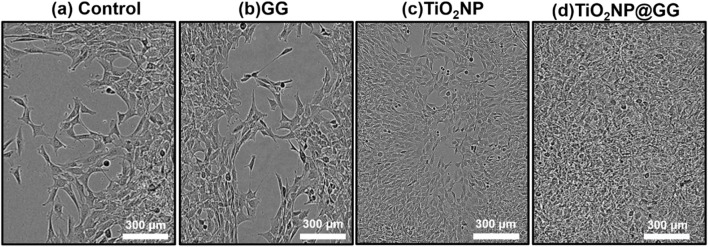
Illustrations of fibroblast cells moving into a scratch area after 24 h for **(a)** control sample and in the presence of **(b)** GG, **(c)** TiO_2_NP, and **(d)** TiO_2_NP@GG hydrogel.

After 24 h of incubation, the control sample of TCPP and GG hydrogel displayed minimal wound closure (Figures 12a, b. In contrast, bare TiO_2_NPs sample exhibited better wound closure performance compared to the TCPP control and GG hydrogel samples (Figure 12c. Conversely, the TiO_2_NP@GG hydrogel exhibited complete closure of the scratch (Figure 12d. These results suggest that the incorporation of TiO_2_NP significantly enhances the rate of fibroblast cell migration and wound healing. The presence of biosynthesized TiO_2_NP in the TiO_2_NP@GG hydrogel promoted cell proliferation and migration, leading to nearly 100% wound closure after 24 h. This is significantly higher compared to the control (∼33%) and GG hydrogel (∼62%) lacking TiO_2_NP. Notably, bare TiO_2_NP alone achieved an intermediate closure rate of approximately 80%, suggesting their inherent bioactivity contributes significantly to the wound healing process. The superior cell migration observed with the TiO_2_NP@GG hydrogel can be attributed to the combined effects of the biosynthesized TiO_2_NP which are their free radical scavenging activity and reduced cytotoxicity (Haghighi et al., 2023). Additionally, the enhanced antibacterial properties associated with TiO_2_NP contribute to preventing bacterial infection and promoting overall wound healing. Table 5 shows the comparison of TiO_2_NP@GG hydrogel performance with various biomaterial dressings loaded with TiO_2_NP for applications in skin tissue engineering and wound healing.

**TABLE 5:** Comparison study of TiO_2_NP@GG hydrogel performance with various biomaterial dressings loaded with TiO_2_NP for applications in skin tissue engineering and wound healing.

Samples	Product formed	Results	Ref.
TiO_2_NP@Gelatin	Gel	- Antibacterial activity against *S. aureus* - Promoted wound contraction	Javanmardi et al. (2018)
TiO_2_NP@Bacteria cellulose	Film	- Antibacterial activity against *E. coli* - Excellent cell growth and proliferation	Khan et al. (2015)
TiO_2_NP@Chitin + silk fibroin	Scaffold	- Antimicrobial activity against *S. aureus*, and *E. coli* - Good cytocompatibility	Mehrabani et al. (2018)
TiO_2_NP@Chitosan + PPG	Hydrogel film	- Enhanced tensile strength and elongation at break - Antimicrobial activity against *S. aureus*, and *E. coli*	Ulu et al. (2020)
TiO_2_NP/CeO_2_@Chitosan/PCL	Scaffold	- Higher tensile strength - Good cell compatibility - Antibacterial activity against *E. coli* and *S. aureus* - Accelerated wound closure	Sanmugam et al. (2024)
TiO_2_NP@Heparin + PVA	Hydrogel	- Improved tensile strength, elongation to break, and Young’s modulus - Antibacterial activity against *E. coli* and *S. aureus* - Enhanced cell proliferation *in vitro* - Accelerated wound closure	Li et al. (2021)
TiO_2_NP@GG	Hydrogel	- Improved tensile strength, elongation to break, and Young’s modulus - Antibacterial activity against *E. coli* and *S. aureus* - 100% wound closure after 24 h	This study

## Conclusion

This study reports the development of a novel TiO_2_NP@GG hydrogel for potential applications in wound healing. The synthesized hydrogel was characterized using XRD, SEM, EDS, and XPS to evaluate its chemical and physical properties. XRD analysis confirmed the successful fabrication of the targeted TiO_2_NP@GG hydrogel, evidenced by the presence of anatase TiO_2_ and amorphous GG in the diffraction pattern. Notably, SEM revealed a non-homogeneous distribution of TiO_2_NP on the hydrogel scaffold, which increased the surface roughness, potentially improving its performance as a wound dressing material. *In vitro* wound healing assays demonstrated that the incorporation of biosynthesized TiO_2_NP promoted cell proliferation and accelerated cell migration in scratch wound models. Additionally, the inherent antibacterial properties of TiO_2_NP further contributed to the favourable performance of the TiO_2_NP@GG hydrogel as a wound dressing material.

## Data Availability

The raw data supporting the conclusions of this article will be made available by the authors, without undue reservation.
